# Association of serum antibodies against the *Mycobacterium avium* complex and hemoptysis: a cross-sectional study

**DOI:** 10.1186/s12879-021-06182-9

**Published:** 2021-05-26

**Authors:** Hiroaki Ogata, Atsushi Moriwaki, Taisuke Nakagawa, Soichiro Sakoda, Akiko Ishimatsu, Kazuhito Taguchi, Hiroshi Aso, Hiroko Nogami, Masako Kadowaki, Yuko Tateshi, Makoto Yoshida

**Affiliations:** 1 Department of Respiratory Medicine, National Hospital Organization Fukuoka National Hospital, 4-39-1 Yakatabaru, Minami-ku, Fukuoka, 811-1394 Japan; 2 Department of Infectious Diseases, National Hospital Organization Fukuoka National Hospital, 4-39-1 Yakatabaru, Minami-ku, Fukuoka, 811-1394 Japan; 3 Department of Radiology, National Hospital Organization Fukuoka National Hospital, 4-39-1 Yakatabaru, Minami-ku, Fukuoka, 811-1394 Japan

**Keywords:** Nontuberculous mycobacteria, *Mycobacterium avium* complex, Hemoptysis, Serum anti-MAC antibody

## Abstract

**Background:**

Hemoptysis is very common and can be life threatening in clinical practice for nontuberculous mycobacteria. The serum antibody against the *Mycobacterium avium* complex (MAC-Ab), the majority of nontuberculous mycobacteria species, is well known to reflect the activity of MAC lung disease; however, there is no study investigating the association between the MAC-Ab and hemoptysis in MAC patients. Therefore, we assessed whether the MAC-Ab is a good biomarker for hemoptysis among subjects with MAC lung disease.

**Methods:**

This study was conducted as a five-year retrospective survey at the National Hospital Organization Fukuoka National Hospital. A total of 155 patients aged ≥20 years with MAC lung disease were enrolled and separated into seropositive and seronegative groups using the cutoff for MAC-Ab levels of 0.7 U/ml. The prevalence of hemoptysis and odds ratios for the presence of hemoptysis were estimated and compared between the groups. To investigate the linear trends in the relationship between MAC-Ab levels and hemoptysis, the subjects were classified into three groups using the tertile distribution of the MAC-Ab.

**Results:**

The prevalence of hemoptysis was twice as high in the seropositive group than in the seronegative group (42.2 and 21.7%, respectively, *P* = 0.02). The multivariable-adjusted risk of hemoptysis was elevated in the seropositive group as compared with the seronegative group (odds ratio = 2.79 (95% confidence interval 1.15–7.44)). Likewise, when categorizing the subjects into three groups, the risk of hemoptysis increased with increasing MAC-Ab levels (*P* = 0.03 for trend).

**Conclusions:**

A positive MAC-Ab level was a significant risk factor for hemoptysis among patients with MAC lung disease. There were also positive trends in the association between the MAC-Ab titer and the likelihood of hemoptysis. Measuring the MAC-Ab may contribute not only to early detection of the risk of hemoptysis but also to early intervention with anti-NTM therapy and, as a result, to the prevention of hemoptysis in MAC patients.

**Supplementary Information:**

The online version contains supplementary material available at 10.1186/s12879-021-06182-9.

## Background

Hemoptysis is one of the most common symptoms of nontuberculous mycobacteria (NTM) diseases. The prevalence of hemoptysis has been estimated to be 16.7–42.6% among NTM cases, indicating that it is a commonplace event regardless of the geographic or clinical characteristics of study populations [1–4]. In addition, it is a potentially life-threatening condition and known as a predictor of poor prognosis and mortality [5], and thus NTM patients with hemoptysis tend to be treated with antibiotic agents [6, 7]. With the worldwide increase in the prevalence of NTM infection [5, 8–12], the identification of risk factors for hemoptysis will contribute to the establishment of effective management of NTM, such as raising awareness, making decisions about early intervention with anti-NTM therapy, and perhaps the prediction of morbidity and mortality.

In clinical practice for *Mycobacterium avium* complex (MAC) lung disease, consisting largely of NTM [5, 6, 8, 11], the serum antibody against MAC (MAC-Ab) is a strong serodiagnostic tool whose sensitivity and specificity have been reported to be as high as 78.6–92.3% and 96.4–100%, respectively [13–15]. In addition to its reliability, MAC-Ab measurement is a rapid, noninvasive, and easy-to-use test [16]. Although it is reported to reflect disease activity [13, 14], there has been no study evaluating its association with various clinical presentations, including hemoptysis, among patients with MAC disease. Thus, investigating this matter is of great benefit for improving the management of such patients.

Based on these considerations, the present study was conducted as a five-year survey in order to evaluate the associations between the MAC-Ab and hemoptysis using subjects with MAC lung disease.

## Methods

### Study population

**Fig. 1:**
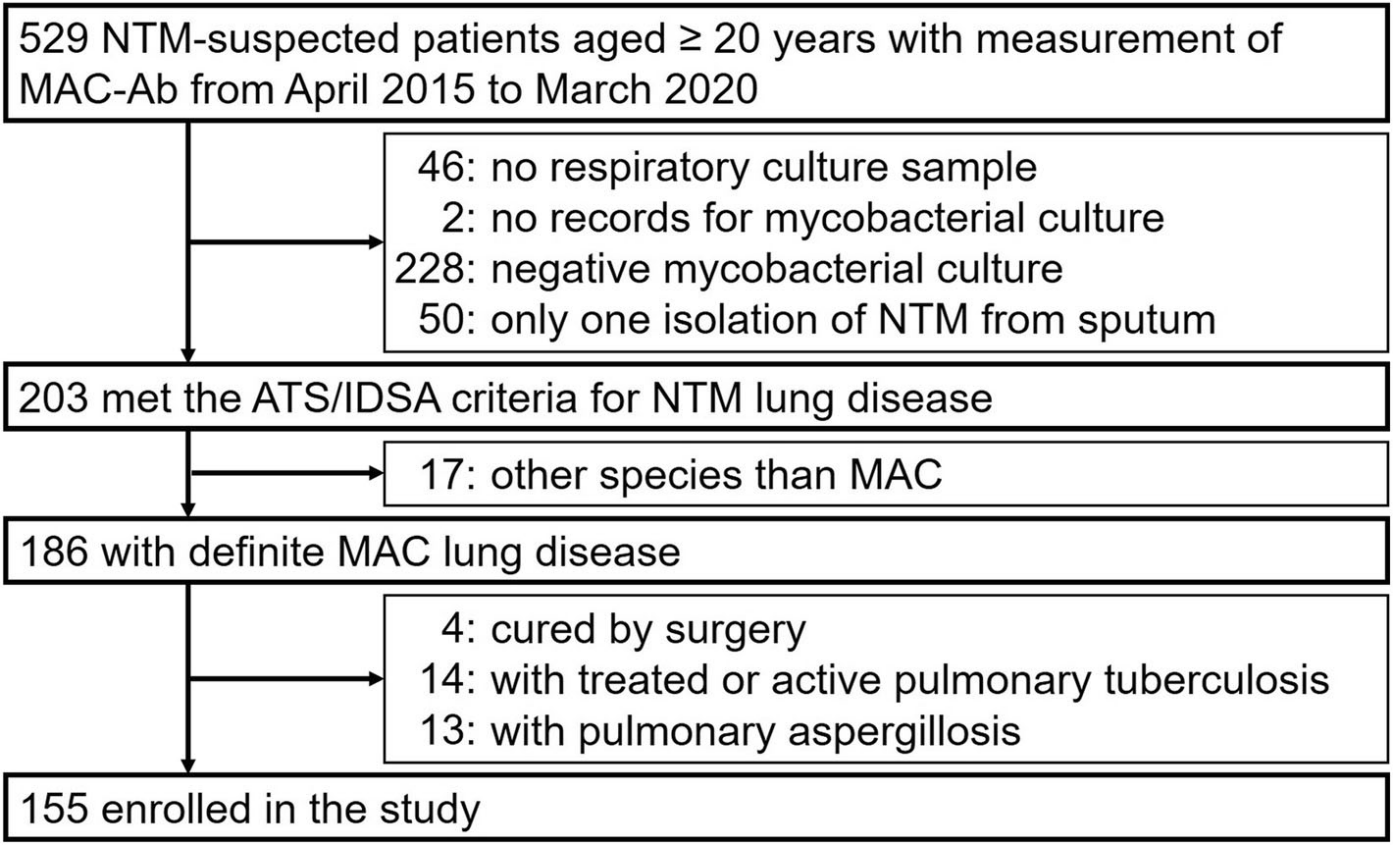
Selection of subjects

This study was conducted through a retrospective review of medical records at National Hospital Organization Fukuoka National Hospital. We reviewed 529 patients aged ≥20 years who were clinically suspected to be NTM positive and measured their MAC-Ab titer between April 1, 2015, and March 31, 2020. Among them, we excluded 46 patients who were either unable or unwilling to provide respiratory culture samples, 2 patients with no available medical records for mycobacterial culture, 228 patients whose mycobacterial cultures were negative, and 50 patients with only a single incident of NTM isolation from the sputum specimen culture; the remaining 203 patients met the American Thoracic Society / Infectious Diseases Society of America (ATS/IDSA) diagnostic criteria for NTM lung disease [6]. There were 17 cases whose identified species of NTM were other than MAC, and 4 cases were cured by surgery; the number of patients with definite MAC lung disease was 186. Since pulmonary tuberculosis and aspergillosis are major infectious diseases that, as well as NTM, can cause hemoptysis [17], 14 patients with treated or active pulmonary tuberculosis and 13 patients with coexisting pulmonary aspergillosis were also excluded. Hence, a total of 155 cases of MAC lung disease were enrolled in the present study (Fig. 1).

### MAC-ab assessment

The MAC-Ab was measured as a serum immunoglobulin A antibody against the glycopeptidolipid core using the enzyme-linked immunosorbent assay kit (Tauns Laboratory Inc., Shizuoka, Japan) according to the manufacturer’s instructions. Data were expressed as U/ml in relation to the standard curve generated using reference sera. Technicians without prior knowledge of the clinical data performed the assay. In accordance with previous reports [13–15], we defined seropositive and seronegative MAC as cases with MAC-Ab levels of ≥0.7 U/ml and <  0.7 U/ml, respectively. When dividing the study subjects into three groups based on the tertile distribution of MAC-Ab titers, the cutoff values were as follows: lowest, ≤ 0.9 U/ml; middle, 1.0–4.4 U/ml; and highest, ≥ 4.5 U/ml.

### Clinical evaluations

For each case, respiratory physicians reviewed the patient’s medical records and assessed the demographic and clinical characteristics: age, gender, height, weight, medical history, results of mycobacterial cultures, antibiotic treatment for MAC disease, and use of anticoagulant and/or antiplatelet agents. Body mass index (BMI; kg/m^2^) was calculated as weight divided by squared height. Hemoptysis was defined as the presence of fresh blood in the sputum and/or spit, with confirmation by respiratory physicians. Together with a radiologist, respiratory physicians also interpreted the chest computed tomography (CT) images of each case. According to the official ATS/IDSA statement for CT findings of NTM [6], the subjects were classified as having nodular bronchiectatic disease, fibrocavitary disease, or other diseases (i.e., solitary nodular disease, hypersensitivity-like disease, and disseminated disease).

### Statistical analysis

SAS University Edition software version 9.4 (SAS Institute, Cary, NC, USA) was used to perform all statistical analyses. A two-sided *P* <  0.05 was considered to indicate statistical significance. Demographic and clinical characteristics were compared between the seropositive and seronegative groups using an analysis of variance for continuous variables or a Chi-squared test for dichotomous or categorical ones. The frequency of hemoptysis was calculated separately for MAC-Ab presence. The differences in the frequency of hemoptysis between the seropositive and seronegative groups were assessed using a Chi-squared test. The association of the MAC-Ab with hemoptysis was estimated as an odds ratio (OR) with 95% confidence intervals (95% CIs) in a multivariable-adjusted logistic regression model, wherein adjustment was made for age, gender, BMI, nodular bronchiectatic disease, fibrocavitary disease, and the number of NTM species identified. The same model was used to evaluate multivariable-adjusted ORs with 95% CIs for each of the covariates. To assess the linear trends in the relationship between MAC-Ab levels and the risk of hemoptysis, we divided the subjects into three groups using the tertile distribution of the MAC-Ab, as described above. The robustness of the main results was tested through sensitivity analyses using only the patients without anticoagulant and/or antiplatelet medication, or only the antibiotic treatment–naïve ones. We also conducted analyses limiting the subjects to those whose culture samples were positive for *Mycobacterium avium* (*M. avium*) or *Mycobacterium intracellulare* (*M. intracellulare*) individually.

### Ethical considerations

The study was approved by the National Hospital Organization Fukuoka National Hospital Institutional Review Board for Clinical Research (#F2–5).

## Results

### Demographic and clinical characteristics

Table 1 lists the demographic and clinical characteristics according to the presence of the MAC-Ab. Compared with seronegative subjects, seropositive ones were less likely to be male. As to other variables (i.e., age, BMI, distribution of NTM species, CT findings, antibiotic treatment, and anticoagulant and/or antiplatelet therapy), there were no significant differences between the two groups.

**Table 1:** Mean values or frequencies of demographic and clinical characteristics according to MAC-Ab presence

Variables	Seronegative group	Seropositive group	*P* value
	(*n* = 46)	(*n* = 109)	
Male gender (%)	43.5	18.3	0.001
Age (years)	72 (10)	73 (11)	0.61
BMI (kg/m^2^)	19.7 (3.0)	18.6 (3.1)	0.06
NTM species			
^a^*Mycobacterium avium* (%)	58.7	73.4	0.07
^a^*Mycobacterium intracellulare* (%)	50.0	35.8	0.10
Isolation of multiple species of NTM (%)	23.9	13.8	0.12
Appearance on computed tomography			
Nodular bronchiectatic disease (%)	80.4	70.6	0.10
Fibrocavitary disease (%)	17.4	29.4	
Others (%)	2.2	0.0	
Antibiotic treatment for MAC disease (%)	54.3	61.5	0.41
Anticoagulant and/or antiplatelet medication (%)	13.0	5.5	0.11

The seropositive and seronegative groups were defined as the subjects with MAC-Ab levels of ≥0.7 U/ml and < 0.7 U/ml, respectively. Values are given as mean with standard deviations in brackets for continuous variables and as percentages for dichotomized and categorical variables. *P* value denotes the statistical significance of the difference in each variable between seronegative and seropositive groups

*MAC-Ab* antibody against the *Mycobacterium avium* complex, *BMI* body mass index, *NTM* nontuberculous mycobacteria

^a^Patients from whom both *Mycobacterium avium* and *Mycobacterium intracellulare* were isolated (*n* = 14) were included

### Associations of the MAC-ab with hemoptysis

**Fig. 2:**
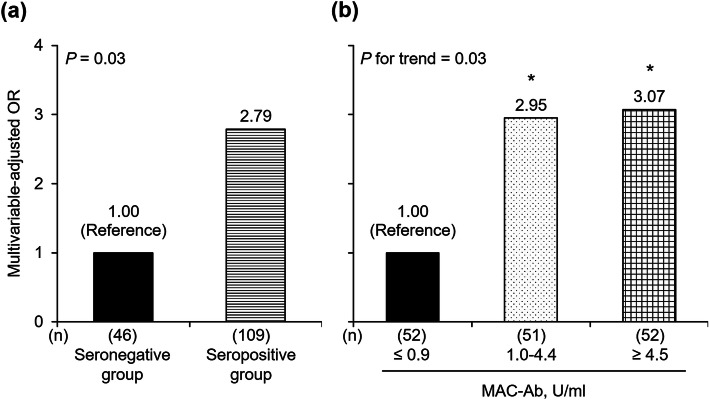
Trends in the multivariable-adjusted odds ratios for hemoptysis according to MAC-Ab levels. OR, odds ratio; MAC-Ab, antibody against the *Mycobacterium avium* complex. ^*^P < 0.05 versus the reference group. **a** The seropositive and seronegative groups were defined as the subjects with MAC-Ab levels of ≥0.7 U/ml and < 0.7 U/ml, respectively. **b** The study subjects were divided into three groups based on the tertile distribution of MAC-Ab titers as follows: lowest (reference), < 0.9 U/ml; middle, 1.0–4.4 U/ml; and highest, ≥ 4.5 U/ml. In both **a** and **b**, adjustments were made for age, gender, BMI, nodular bronchiectatic disease, fibrocavitary disease, and the number of NTM species identified

There were 56 subjects with hemoptysis (36.1% of the total study subjects). The prevalence of hemoptysis was twice as high in the seropositive group than in the seronegative group (42.2 and 21.7%, respectively, *P* = 0.02). After adjustment for potential confounders, the positive association of the MAC-Ab with hemoptysis remained significant (OR = 2.79 (95% CI 1.15–7.44), *P* = 0.03) (Fig. 2a). The other variables were not associated with hemoptysis (all *P* ≥ 0.14) (Table 2). When classifying the subjects into three groups, the prevalence of hemoptysis increased gradually with higher MAC-Ab levels (*P* = 0.03 for trend). The sensitivity and specificity at the cutoff values were, respectively, 80.4 and 41.4% at the value of 1.0 U/ml and 39.3 and 69.7% at 4.5 U/ml; the area under the receiver operating characteristic curve was therefore estimated as 0.602 (Additional file 1, Supplementary Fig. S1). In comparison with the lowest tertile group, the multivariable-adjusted ORs for hemoptysis were significantly higher in both the middle and highest tertile groups (OR = 2.95 (1.11–8.32), *P* = 0.03, and OR = 3.07 (1.19–8.52), *P* = 0.02, respectively) (Fig. 2b).

**Table 2:** Multivariable-adjusted odds ratios of potential risk factors for hemoptysis

Risk factors	Number of hemoptysis events/cases (%)		Multivariable-adjusted OR (95% CI)	*P* value
	Exposure group	Reference group		
MAC-Ab positive (vs. negative)	46/109 (42.2%)	10/46 (21.7%)	2.79 (1.15–7.44)	0.03
Age (per 1-year increase)	N/A	N/A	0.99 (0.96–1.03)	0.62
Male gender (vs. female)	8/40 (20.0%)	48/115 (41.7%)	0.50 (0.19–1.22)	0.14
BMI (per 1 kg/m^2^ increase)	N/A	N/A	0.96 (0.85–1.09)	0.52
Nodular bronchiectatic disease (vs. others)	42/114 (36.8%)	14/41 (34.1%)	> 999.99 (0.02- > 999.99)	0.96
Fibrocavitary disease (vs. others)	14/40 (35.0%)	42/115 (36.5%)	> 999.99 (0.01- > 999.99)	0.96
Number of NTM species (per increase of 1)	N/A	N/A	1.87 (0.75–4.61)	0.17

Adjustment was made for MAC-Ab presence, age, gender, BMI, nodular bronchiectatic disease, fibrocavitary disease, and the number of NTM species identified

*OR* odds ratio, *95% CI* 95% confidence interval, *MAC-Ab* antibody against the *Mycobacterium avium* complex, *N/A* not applicable, *BMI* body mass index, *NTM* nontuberculous mycobacteria

The prevalence of hemoptysis in *M. avium*–identified cases was almost the same as in *M. intracellulare*–identified ones (38.3 and 37.1%, respectively). Although not statistically significant, a similar trend of relationship between the MAC-Ab and hemoptysis was observed both in *M. avium*–positive and *M. intracellulare*–positive cases (OR = 2.35 (0.75–8.55), *P* = 0.16, and OR = 3.12 (0.86–13.13), *P* = 0.10, respectively) (Additional file 1, Supplementary Fig. S2). In addition, the results were substantially similar when limiting the subjects to those without anticoagulant / antiplatelet medication (Additional file 1, Supplementary Fig. S3) or treatment-naïve ones (Additional file 1, Supplementary Fig. S4).

## Discussion

This retrospective study revealed that detection of the MAC-Ab was a significant risk factor for hemoptysis among patients with MAC pulmonary disease. The significant positive trends were also observed in the association of the MAC-Ab titer with the prevalence of hemoptysis. As with the species-specific analyses, neither the sensitivity analyses among patients with no anticoagulant / antiplatelet agents nor those among the patients with no NTM treatment altered the results substantially. This is the first study to evaluate the influence of disease activity, based on the MAC-Ab, on hemoptysis in NTM patients.

Hemoptysis is well known to be progressively more prevalent with advancing NTM lung disease [6]. Since the MAC-Ab titer is reported to reflect disease activity [13, 14], it is considered to be a biomarker for disease progression and, as a consequence, hemoptysis. Since hemoptysis is one of the most principal factors leading physicians to initiate NTM treatment [6, 7], measuring the MAC-Ab may contribute not only to the early detection of high-risk patients for hemoptysis but also to early intervention in NTM and, as a result, to the prevention of hemoptysis in MAC patients. Given the relation of hemoptysis to poor prognosis among NTM patients [5], MAC-Ab measurement might even help with the prediction of morbidity and mortality in those populations.

To date, there has been only one study investigating risk factors for hemoptysis in subjects with NTM lung disease that mentioned that there was no significant biomarker for hemoptysis except for being young [3]. The inverse association between age and hemoptysis was assumed to be due to age-related decline in immune responses [3, 18]; the negative impact of age might have been by virtue of an interaction with immune activation against NTM pathogens. Meanwhile, with regard to tuberculosis, the effects of age on hemoptysis are controversial [18–20]. In the present study, testing positive for the MAC-Ab was significantly related to an increase in the prevalence of hemoptysis, while being young was not; the MAC-Ab seemed to be a more valuable marker for the progression of MAC disease and hemoptysis than age. As for the impact of age on hemoptysis, further research is warranted.

Regarding MAC species, both the frequency of hemoptysis and the impact of the MAC-Ab on hemoptysis were equivalent between subjects with detected *M. avium* and *M. intracellulare* in this study. The former findings were in line with previous research [3, 21]. Moreover, there was no evidence of differences in MAC-Ab levels between subjects with *M. avium* and those with *M. intracellulare* [13, 14]. This suggests that, in patients with MAC lung disease, the MAC-Ab is a universal risk factor for hemoptysis even in the population with a different distribution of MAC species from ours.

The strengths of our study were the highly accurate diagnosis of NTM based on the ATS/IDSA criteria and the exclusion of cases with coexisting pulmonary tuberculosis or aspergillosis to minimize factors perturbing the relationship between the MAC-Ab and hemoptysis. However, some potential limitations should be noted. First, the MAC-Ab levels were based on a single measurement. This may cause misclassification of the MAC-Ab level, which could have weakened the association found in the present study, biasing the results toward a null hypothesis. Second, there was the possibility of an increase in the risk of hemoptysis due to anticoagulant/antiplatelet use. However, there was no significant difference in the frequency of such medication use between the seropositive and seronegative groups. Additionally, the sensitivity analyses excluding subjects with such medication showed similar results. Thus, this potential bias did not appear to have affected the present results. Third, antibiotic treatment might have reduced the risk of hemoptysis in a part of the study population and consequently weakened the results. In our study, the proportion of subjects with NTM treatment was, to some extent, higher in the seropositive group than in the seronegative one; the positive association of the MAC-Ab with hemoptysis might have been underestimated. However, similar findings were observed in the analyses including only patients with no NTM treatment. Therefore, this limitation may not have altered our conclusions. Fourth, we did not have access to pulmonary function data to assess the disease severity of each case. However, as for NTM cases, there was no association between treatment outcomes and pulmonary function changes [22]. Hence, pulmonary function seems not to have affected or interacted the present outcomes. Fifth, the present outcomes might lack external validity and generalizability owing to the study design as single-center analyses. However, the prevalence of hemoptysis in the present study was in accord with previous epidemiological evidence (about one-third of NTM patients) [1–4]. Furthermore, as mentioned above, the results were consistent across MAC species; we speculate that it is reasonable to extrapolate the current findings to the general population with MAC infection. Lastly, the study design adopted in the present investigation, i.e., a cross-sectional design, is not ideal for predicting the future risk of hemoptysis. A prospective cohort study observing the onset of hemoptysis after serum MAC antibody measurement in patients with no history of hemoptysis is needed. To overcome the aforementioned limitations and verify the present results, planning for a multicenter prospective cohort study is underway.

## Conclusions

Testing positive for the MAC-Ab was a significant risk factor for hemoptysis among MAC lung disease patients. An increase in the MAC-Ab titer was associated with a higher likelihood of hemoptysis. Our findings suggested that MAC patients with higher MAC-Ab levels should be considered at high risk for hemoptysis and should be carefully observed for the potential development of that condition in clinical practice. Currently, it is unknown whether or to what extent reducing the MAC-Ab levels can attenuate the risk of hemoptysis. A clinical trial is needed to clarify whether NTM treatment reduces the risk of developing hemoptysis in parallel with a decrease in the MAC-Ab titer.

## Supplementary Information

**Additional file 1: Supplementary Figure S1.** The receiver operating characteristic curve for the diagnostic accuracy of MAC-Ab levels. **Supplementary Figure S2.** Multivariable-adjusted odds ratios for hemoptysis according to MAC-Ab presence, separately, for MAC species. **Supplementary Figure S3.** Multivariable-adjusted odds ratios for hemoptysis according to MAC-Ab presence among the patients with MAC lung disease and without anticoagulant and/or antiplatelet medication. **Supplementary Figure S4.** The prevalence of hemoptysis according to MAC-Ab presence among treatment-naïve patients with MAC lung disease.

## Data Availability

The datasets used and/or analysed during the current study are available from the corresponding author on reasonable request.

## Abbreviations

ATS/IDSA: the American Thoracic Society / Infectious Diseases Society of America
BMI: Body mass index
CT: Computed tomography
*M. avium*: *Mycobacterium avium*
MAC: *Mycobacterium avium* complex
MAC-Ab: Serum antibody against the *Mycobacterium avium* complex
*M. intracellulare*: *Mycobacterium intracellulare*
NTM: Nontuberculous mycobacteria
OR: Odds ratio
95% CIs: 95% confidence intervals

## References

[1] Klann E, Beal SG, Tremblay EE (2019). Evaluating differences in tuberculosis and nontuberculous mycobacterial lung disease in Florida. Am J Infect Control.

[2] Hu C, Huang L, Cai M, Wang W, Shi X, Chen W (2019). Characterization of non-tuberculous mycobacterial pulmonary disease in Nanjing district of China. BMC Infect Dis.

[3] Lee SH, Lee JH, Chang JH, Kim SJ, Yoon H-Y, Shim SS, Kim MU, Choi SY, Ryu YJ (2019). Hemoptysis requiring bronchial artery embolization in patients with nontuberculous mycobacterial lung disease. BMC Pulm Med.

[4] Zhang ZX, Cherng BPZ, Sng LH, Tan YE (2019). Clinical and microbiological characteristics of non-tuberculous mycobacteria diseases in Singapore with a focus on pulmonary disease, 2012–2016. BMC Infect Dis.

[5] Cowman S, van Ingen J, Griffith DE, Loebinger MR (2019). Non-tuberculous mycobacterial pulmonary disease. Eur Respir J.

[6] Griffith DE, Aksamit T, Brown-Elliott BA, Catanzaro A, Daley C, Gordin F, Holland SM, Horsburgh R, Huitt G, Iademarco MF, Iseman M, Olivier K, Ruoss S, von Reyn C, Wallace RJ Jr, Winthrop K, ATS Mycobacterial Diseases Subcommittee, American Thoracic Society, Infectious Disease Society of America (2007). An official ATS/IDSA statement: diagnosis, treatment, and prevention of nontuberculous mycobacterial diseases. Am J Respir Crit Care Med.

[7] Provoost J, Valour F, Gamondes D, Roux S, Freymond N, Perrot E, Souquet PJ, Kiakouama-Maleka L, Chidiac C, Lina G, Dumitrescu O, Sénéchal A, Ader F (2018). A retrospective study of factors associated with treatment decision for nontuberculous mycobacterial lung disease in adults without altered systemic immunity. BMC Infect Dis.

[8] Morimoto K, Iwai K, Uchimura K, Okumura M, Yoshiyama T, Yoshimori K, Ogata H, Kurashima A, Gemma A, Kudoh S (2014). A steady increase in nontuberculous mycobacteriosis mortality and estimated prevalence in Japan. Ann Am Thorac Soc.

[9] Adjemian J, Olivier KN, Seitz AE, Holland SM, Prevots DR (2012). Prevalence of nontuberculous mycobacterial lung disease in U.S. Medicare beneficiaries. Am J Respir Crit Care Med.

[10] Prevots DR, Marras TK (2016). Epidemiology of human pulmonary infection with non- tuberculous mycobacteria: a review. Clin Chest Med.

[11] Shah NM, Davidson JA, Anderson LF, Lalor MK, Kim J, Thomas HL, Lipman M, Abubakar I (2016). Pulmonary *Mycobacterium avium-intracellulare* is the main driver of the rise in non-tuberculous mycobacteria incidence in England, Wales and Northern Ireland, 2007–2012. BMC Infect Dis.

[12] Ding LW, Lai CC, Lee LN, Hsueh PR (2006). Disease caused by non-tuberculous mycobacteria in a university hospital in Taiwan, 1997–2003. Epidemiol Infect.

[13] Kitada S, Maekura R, Toyoshima N, Fujiwara N, Yano I, Ogura T, Ito M, Kobayashi K (2002). Serodiagnosis of pulmonary disease due to *Mycobacterium avium* complex with an enzyme immunoassay that uses a mixture of glycopeptidolipid antigens. Clin Infect Dis.

[14] Kitada S, Kobayashi K, Ichiyama S, Takakura S, Sakatani M, Suzuki K, Takashima T, Nagai T, Sakurabayashi I, Ito M, Maekura R, MAC Serodiagnosis Study Group (2008). Serodiagnosis of *Mycobacterium avium*–complex pulmonary disease using an enzyme immunoassay kit. Am J Respir Crit Care Med.

[15] Kitada S, Kobayashi K, Nishiuchi Y, Fushitani K, Yoshimura K, Tateishi Y, Miki K, Miki M, Hashimoto H, Motone M, Fujikawa T, Hiraga T, Maekura R (2010). Serodiagnosis of pulmonary disease due to *Mycobacterium avium* complex proven by bronchial wash culture. Chest..

[16] Kobayashi K (2014). Serodiagnosis of *Mycobacterium avium* complex disease in humans: translational research from basic mycobacteriology to clinical medicine. Jpn J Infect Dis.

[17] Davidson K, Shojaee S (2020). Managing massive hemoptysis. Chest..

[18] Achkar JM, Joseph G (2012). Independent association of younger age with hemoptysis in adults with pulmonary tuberculosis. Int J Tuberc Lung Dis.

[19] Hwang HG, Lee HS, Choi JS, Seo KH, Kim YH, Na JO (2013). Risk factors influencing rebleeding after bronchial artery embolization on the management of hemoptysis associated with pulmonary tuberculosis. Tuberc Respir Dis (Seoul).

[20] Katoto PDMC, Murhula A, Kayembe-Kitenge T, Lawin H, Bisimwa BC, Cirhambiza JP, Musafiri E, Birembano F, Kashongwe Z, Kirenga B, Mfinanga S, Mortimer K, de Boever P, Nawrot T, Nachega J, Nemery B (2018). Household air pollution is associated with chronic cough but not hemoptysis after completion of pulmonary tuberculosis treatment in adults, rural eastern Democratic Republic of Congo. Int J Environ Res Public Health.

[21] Obayashi Y, Fujita J, Suemitsu I, Kamei T, Nii M, Takahara J (1998). Clinical features of non-tuberculous mycobacterial disease: comparisons between smear-positive and smear-negative cases, and between *Mycobacterium avium* and *Mycobacterium intracellulare*. Int J Tuberc Lung Dis.

[22] Kobayashi T, Tsuyuguchi K, Arai T, Tsuji T, Maekura T, Kurahara Y, Sugimoto C, Minomo S, Nakao K, Tokura S, Sasaki Y, Hayashi S, Inoue Y, Suzuki K (2018). Change in lung function in never-smokers with nontuberculous mycobacterial lung disease: a retrospective study. J Clin Tuberc Other Mycobact Dis.

