# Supplementary material for: Association of serum antibodies against the Mycobacterium avium complex and hemoptysis: a cross-sectional study
Source: BMC Infect Dis. 2021 May 26;21:480. doi: 10.1186/s12879-021-06182-9 (PMC8157429; doi:10.1186/s12879-021-06182-9)
Supplement: Supplementary file 1 — Additional file 1: Supplementary Figure S1. The receiver operating characteristic curve for the diagnostic accuracy of MAC-Ab levels. Supplementary Figure S2. Multivariable-adjusted odds ratios for hemoptysis according to MAC-Ab presence, separately, for MAC species. Supplementary Figure S3. Multivariable-adjusted odds ratios for hemoptysis according to MAC-Ab presence among the patients with MAC lung disease and without anticoagulant and/or antiplatelet medication. Supplementary Figure S4. The prevalence of hemoptysis according to MAC-Ab presence among treatment-naïve patients with MAC lung disease. [file 12879_2021_6182_MOESM1_ESM.docx]

**Additional file 1**

**Supplementary Figure S1.** The receiver operating characteristic curve for the diagnostic accuracy of MAC-Ab levels.


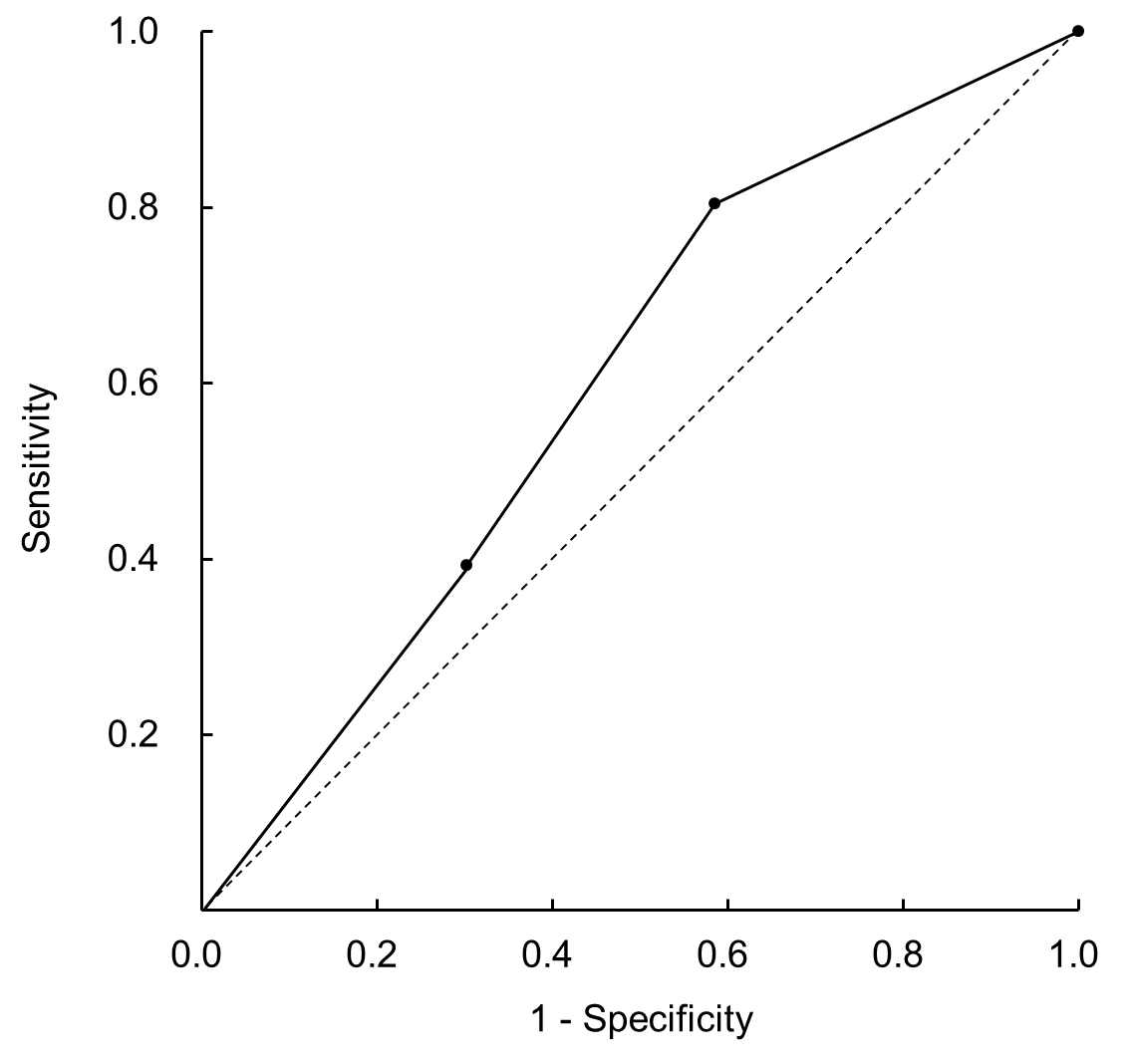


**Supplementary Figure S2.** Multivariable-adjusted odds ratios for hemoptysis according to MAC-Ab presence, separately, for MAC species.


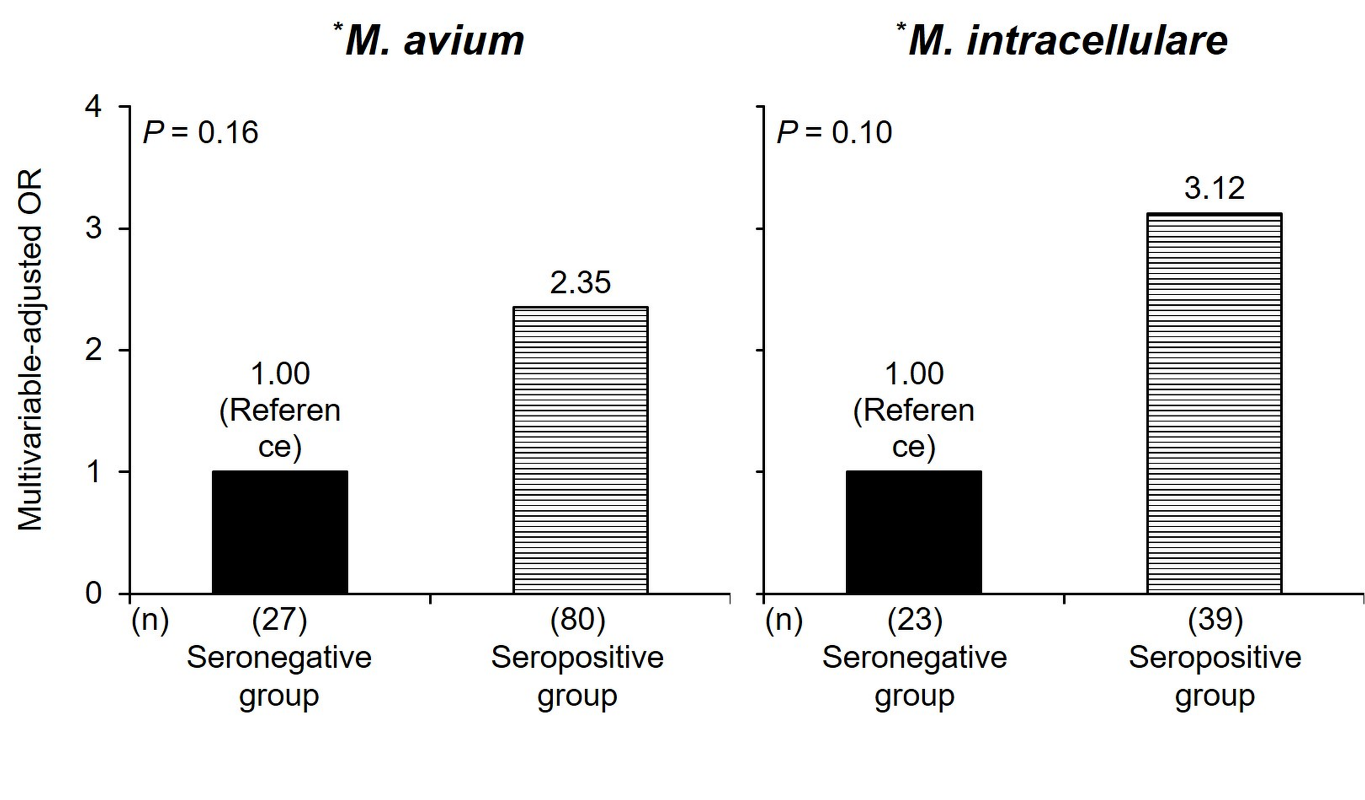


*M. avium*, *Mycobacterium avium*; *M. intracellulare*, *Mycobacterium intracellulare*; OR, odds ratio.

The seropositive and seronegative groups were defined as the subjects with MAC-Ab levels of ≥ 0.7 U/ml and <0.7 U/ml, respectively. Adjustments were made for age, gender, BMI, nodular bronchiectatic disease, fibrocavitary disease, and the number of NTM species identified.

^*^Patients from whom both *Mycobacterium avium* and *Mycobacterium intracellulare* were isolated (n = 14) were included.

**Supplementary Figure S3.** Multivariable-adjusted odds ratios for hemoptysis according to MAC-Ab presence among the patients with MAC lung disease and without anticoagulant and/or antiplatelet medication.


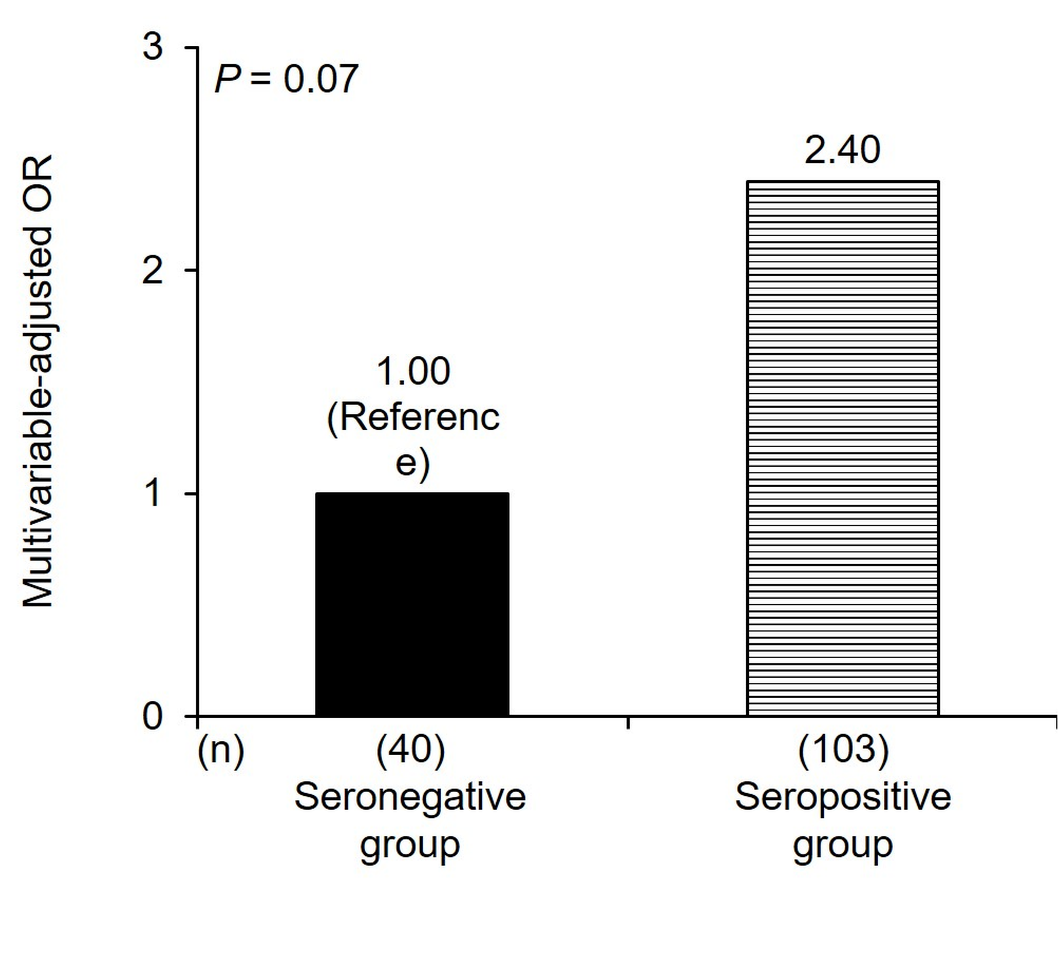


OR, odds ratio.

The seropositive and seronegative groups were defined as the subjects with MAC-Ab levels of ≥ 0.7 U/ml and <0.7 U/ml, respectively. Adjustments were made for age, gender, BMI, nodular bronchiectatic disease, fibrocavitary disease, and the number of NTM species identified.

**Supplementary Figure S4.** The prevalence of hemoptysis according to MAC-Ab presence among treatment-naïve patients with MAC lung disease.


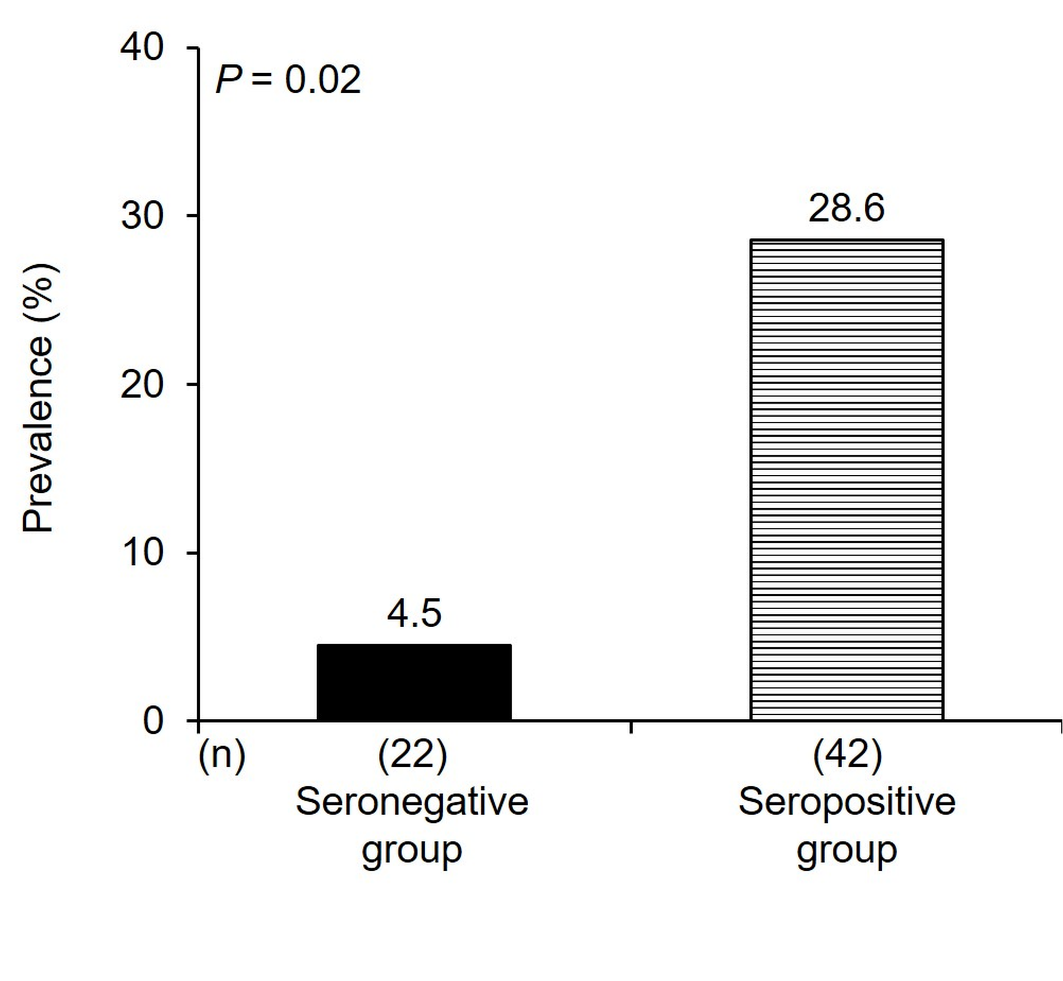


The seropositive and seronegative groups were defined as the subjects with MAC-Ab levels of ≥ 0.7 U/ml and <0.7 U/ml, respectively.
